# Extraordinary optical transmission in silicon nanoholes

**DOI:** 10.1038/s41598-021-01068-x

**Published:** 2021-11-03

**Authors:** Hosam Mekawey, Yehea Ismail, Mohamed Swillam

**Affiliations:** 1 School of Science and Engineering, Center of Nanoelectronics and Devices (CND), The American University in Cairo, New Cairo, Egypt; 2 Department of Physics, School of Science and Engineering, The American University in Cairo, New Cairo, 11835 Egypt

**Keywords:** Mid-infrared photonics, Optical sensors, Metamaterials, Silicon photonics, Nanocavities

## Abstract

In this work, for the first time, a study was conducted of the existence of Extraordinary Optical Transmission (EOT) in Silicon (Si) thin films with subwavelength holes array and high excess carrier concentration. Typically EOT is studied in opaque perforated metal films. Using Si would bring EOT and its many applications to the silicon photonics realm and the mid-IR range. Since Si thin film is a semi-transparent film in mid-IR, a generalization was proposed of the normalized transmission metric used in literature for EOT studies in opaque films. The plasma dispersion effect was introduced into the studied perforated Si film through either doping or carriers’ generation. Careful consideration for the differences in optical response modeling in both cases was given. Full-wave simulation and analysis showed an enhanced transmission when using Si with excess carriers, mimicking the enhancement reported in perforated metallic films. EOT was found in the mid-IR instead of the visible range which is the case in metallic films. The case of Si with generated excess carriers showed a mid-IR EOT peak reaching 157% around 6.68 µm, while the phosphorus-doped Si case showed a transmission enhancement of 152% around 8.6 µm. The effect of varying the holes’ dimensions and generated carriers’ concentration on the transmission was studied. The analogy of the relation between the fundamental mode cutoff and the EOT peak wavelength in the case of Si to the case of metal such as silver was studied and verified. The perforated Si thin film transmission sensitivity for a change in the refractive index of the holes and surroundings material was investigated. Also, a study of the device potential in sensing the hole and surroundings materials that have almost the same refractive index yet with different absorption fingerprints was performed as well.

## Introduction

Studying the interaction of light with nano-sized objects led to the discovery of many phenomena and the emergence of the Nano-photonics field. Research in such a field was accelerated by recent advances in nanofabrication and characterization techniques. Extraordinary Optical Transmission (EOT), discovered while studying light transmission through metal thin films with subwavelength holes array, was among the phenomena receiving much research since its discovery in 1998 ^1^. Studying the transmission of light through perforated screens was conducted historically centuries ago. It was well established that as long as a hole in a perforated screen has dimensions larger than the wavelength of the light, it would transmit all the light incident on it. On the other hand, sub-wavelength holes exhibit very poor transmission according to Bethe’s diffraction theory, founded more than half a century ago ^2^. Bethe’s theory was based on the assumption that the perforated metallic screen is a perfect conductor. Up till recently, due to fabrication limitations in the past, it was not feasible to verify through experimentation the applicability of Bethe’s theory for predicting the transmission through a sub-wavelength holes array in a thin real metal screen. In 1998, EOT was first discovered by Ebbesen et al. ^1^ in such a configuration. It was found that the normalized-to-hole-area transmission was several orders of magnitude larger than what was estimated by Bethe’s theory, reaching even a value greater than one. The physical explanation behind EOT was the target of many researchers since its discovery ^3,4,5,6,7,8^. Gordon et al. and Garcia et al. studied the transmission through a single rectangular hole in real metal films ^9–11^. Gordon et al. demonstrated through numerical simulation that, for metal films, enhanced transmission happens near the cutoff wavelength of the fundamental mode of the waveguide formed by a single rectangular hole. Detailed review articles covering recent research advances in EOT are available for more details ^12–14^. EOT can be exploited in applications such as sensing^15–17^, color filters^18,19^, meta-materials^20–22^, meta-lenses^23^, optical trapping^24^, and enhancement of nonlinear effects, among others ^14^.

In addition to its extensive utilization in the nano-electronics industry^25^, doped Silicon (Si) in particular and doped semiconductors, in general, have been the subject of interest in nano-photonics during the last decade. The main drive for such interest is the possibility for semiconductors with high excess carrier concentration to replace metals in generating plasmonics and other subwavelength optical phenomena ^26^. The controllability of the Si plasma frequency, through the introduced level of excess carriers, is a degree of freedom for tuning the range of operation and performance. Moreover, Si fabrication techniques, developed in the electronics industry, paved the way towards low-cost fabrication and smoother integration of photonic devices into today’s electronic circuits. It was noted that heavily doped Si has the potential of shifting induced plasmonics from visible to the mid-IR spectral range ^27,28^. The mid-IR spectral range has drawn the focus of researchers for a broad range of potential applications ranging from biochemical sensing ^29^ to imaging ^30^ and energy harvesting ^31,32^.

In literature, heavily phosphorus-doped Si of carrier densities reaching 4.9 × 10^20^ cm^−3^ was demonstrated ^33^. While for optical generation of carriers, excitation with ultrafast laser can optically produce a large density of excess carrier and/or temperature. Literature shows that electrons and holes carrier concentration of 7 × 10^20^ cm^−3^ can be realized using picosecond laser annealing ^34–36^. In the work presented here, a density of 1 × 10^20^ cm^−3^ was utilized for both cases of phosphorus doped Si and Si with optically generated excess carriers.

In this research, the existence of EOT was investigated for the first time in a perforated Si thin film with a high carrier concentration. The Si material was modeled carefully to differentiate the case of doping from carrier generation and their effect on the overall optical response of the material. Real-time tuning brought by carrier generation compared to doping was the motivation for its inclusion in this work. To enable studying EOT in perforated Si thin films, we introduce a generalization of the normalized-to-area transmission metric, typically used in literature to show EOT in perforated opaque metallic thin films ^1,3,7,9,12^. Such generalization enables studying EOT in semi-transparent perforated thin films as in the case of Si thin films. The relation between the fundamental mode cutoff and the realized EOT in perforated Si thin film was also verified and investigated for an analogy to its counterpart in perforated metal thin films found in the literature ^9,11^. To investigate the capability of using EOT in Si-based perforated films for sensing, a study of the EOT sensitivity for a refractive index change in the hole and surroundings material was performed. Also, a study was performed of the sensitivity for hole and surroundings materials that have almost the same refractive index yet with different absorption fingerprints.

The second section in this article describes the structure design and simulation method. It also discusses the differences in material dispersion between doped Si and Si with generated excess carriers. The third section shows the results and discussion and the conclusions are given in the fourth section.

## Structure design and material dispersion

**Figure 1:**
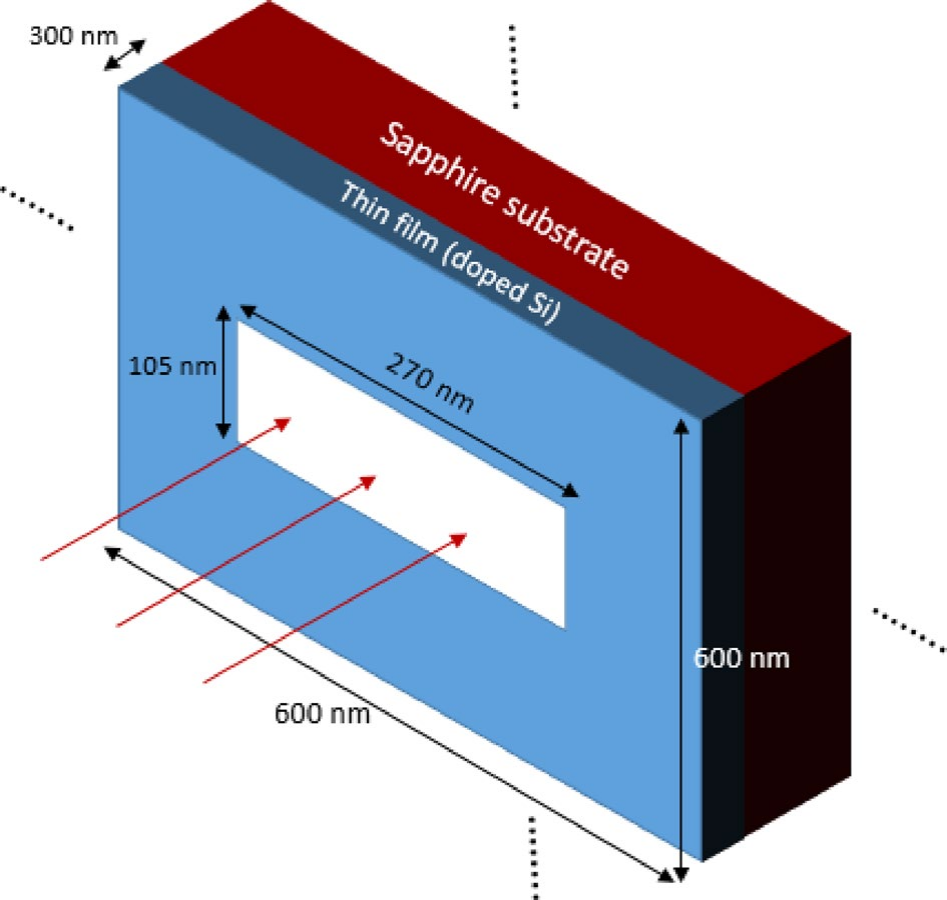
Structure schematics of a periodic cell with a single hole in a thin film. The thin film thickness is 300 nm. The dimensions of the cell are 600 by 600 nm. The dimensions of the hole are 270 by 105 nm. The substrate is sapphire, while the thin film is mainly Si with excess carriers generated or introduced through doping.

The studied structure consists of Si film of 300 nm thickness, perforated with a rectangular holes array. Each hole has dimensions of 270 nm by 105 nm on a sapphire substrate as shown in Fig. 1. The separation between holes’ centers in the holes array is 600 nm in the vertical and horizontal directions. A Finite Difference Time Domain (FDTD) credible simulation package tool ^37^ was used to numerically solve Maxwell equations in 3D and calculate the transmission. The periodic holes array is represented by periodic boundary conditions along the x and y boundaries while an Anisotropic Perfectly Matched Layer (APML) ^38^ boundary condition was used for the z- boundaries where z is the direction of propagation. The film was illuminated in the simulation with an s-polarized plane wave where the E-field vector points along the y-axis.

**Figure 2:**
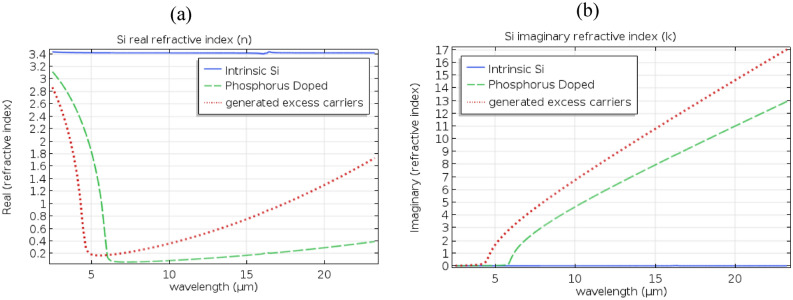
Real and imaginary components of the complex refractive index for intrinsic Si (solid-blue) based on curve fitting of the experimental results by Horowitz et al. and Palik ^39,40^. An index perturbation model was utilized based on soref et al. and henry et al. ^41–43^ to introduce the plasma dispersion effect resulted from a concentration of 10^20^ cm^−3^ free carriers in the case of excess carrier generation (dotted-red) and Phosphorus doping (dashed-green).

In the work presented here, silver, doped Si, and Si with generated excess carriers were used in the structure as the perforated thin-film material at different occasions. Silver material optical response was modeled based on a curve-fitting of Palik’s experimental data^39^. Careful consideration was given to the differences in the optical response between doped Si and Si with optically induced excess carriers. The charge carrier’s mobility would be affected differently in both cases. Doping alters the lattice of the semiconductor by introducing donors or acceptors which affects the scattering cross-section imposed on the excess carriers. Lattice scattering, donor or acceptor scattering, and electron–hole scattering are all factors in the mobility of the excess carriers in this case ^44^. In the case of excess carrier’s concentration, generated optically without doping, the total carrier mobility is affected only by the lattice scattering and the electron–hole scattering effects. The lattice scattering effect on the mobility in the case of nominal doping is represented by the mobility ratio ${{\mu }_{e,h}}_{L}={\mu }_{max}$, where the subscripts *e* and *h* stand for electron and hole respectively and ${\mu }_{max}$ is the maximum mobility value which was found experimentally to be 1417 and 470.5 cm^2^V^−1^ s^−1^ for electron and hole mobilities in Si respectively ^44,45^. More information on ${{\mu }_{e,h}}_{L}$ can be acquired from the unified mobility model presented in ^44^. Also, the electron–hole scattering effect on mobility can be extracted from the same ^44^. In general, Si, with optically generated excess carriers, will show higher mobility values for both electrons and holes compared to the doping case. This is due to the absence of donors or acceptor scatterers. Also, unlike in P or N doping, the fact that the material would have equal high concentrations of electrons and holes in the generated excess carrier case would affect the plasma frequency differently as well. A curve-fitting model was crafted based on the experimental results of both Horowitz et al. ^40^ and Palik ^39^ to provide an accurate optical response model for intrinsic Si in the mid-IR range (from 2.5 µm to 22 µm) (Fig. 2, solid blue curve). This model captures the inter-band transitions and absorption peaks in mid-IR observed in intrinsic Si ^40^. An index perturbation approach was utilized based on the work of Henry et al. ^43^ and Soref et al. ^41,42^ to incorporate the plasma dispersion effect introduced by doping or optical generation of excess carriers into the overall refractive index of the material (Fig. 2 -dashed green and dotted red curves). For 10^20^ cm^−3^ dopant concentration in phosphorus-doped Si, the resulted excess electrons would be 1 × 10^20^ cm^−3^ based on ^46^ where complete ionization is expected above 10^19^ cm^−3^ dopant concentration. The mobility, in this case, would be 79.73 and 162.0 cm^2^V^–1^ s^–1^ for free electrons and free holes respectively based on ^44^ ^45,47,48^. In the case of optically generated excess carriers with 1 × 10^20^ cm^−3^ concentration where there would be no donor/acceptor scattering cross sections in effect, the mobility would be 423.4 and 282.6 cm^2^V^–1^ s^–1^ for free electrons and free holes respectively^44,48^. It was shown in the literature that for concentrations that do not exceed 5 × 10^20^ cm^−3^, the conductivity effective mass can be considered independent from the carrier concentration ^47,49–51^. The effective mass of electron and hole in the work presented here is taken as 0.27m_0_ or 0.37m_0_, respectively^52^. *m*_*0*_ is the free electron mass in a vacuum. The effective mass is assumed to be independent of frequency in the frequency range of interest ^47^.

Figure 2 shows the material dispersion of Si in the cases of phosphorus doping and generated excess carriers for the concentration of 10^20^ cm^−3^ as compared to intrinsic crystalline Si. The dip noticed in the real component of the refractive index corresponds to the crossing of the permittivity from positive to negative indicating the possession of a metallic nature mandatory for inducing Surface Plasmon Polariton (SPP) ^53^. In the case of doping, there is a redshift in the dispersion compared to excess carrier generation as the real part of the dielectric function crosses to a negative value at a higher wavelength in doped Si than in Si with generated carriers.

Sapphire material was utilized in the substrate and its optical response was modeled based on Palik’s experimental data ^39^. The following materials were also used as the holes and surrounding material: Ethane, Methane, and Ethylene. The complex refractive indices of such gases were calculated using Kramers–Kronig relations ^54^ from the transmission data with a 5 cm path length. The transmission data was provided by the NIST chemistry webbook ^55^.

## Results and discussion

**Figure 3:**
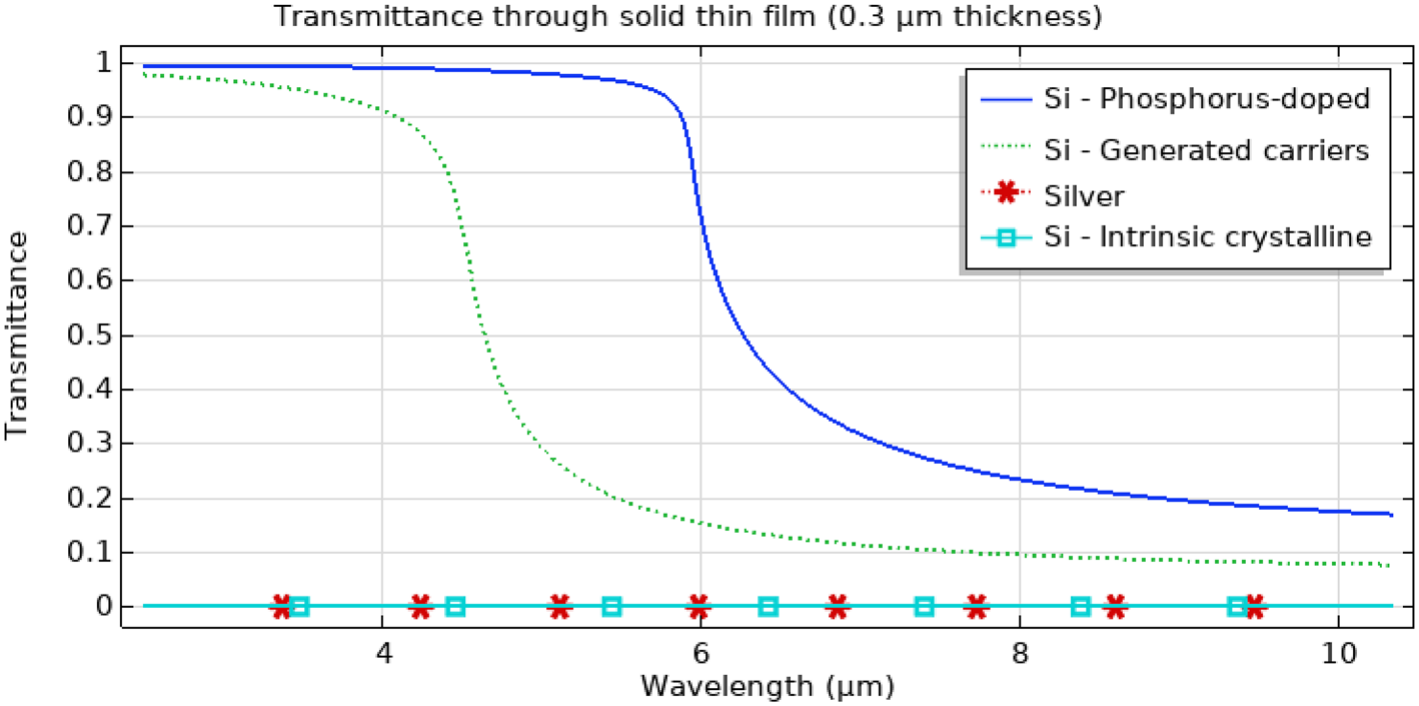
Transmittance as an indication of the opacity of a solid thin film with a thickness of 0.3 µm for Silver, intrinsic crystalline silicon, silicon with 10^20^ cm^−3^ phosphorus doping, and 10^20^ cm^−3^ generated excess carrier concentration. While silver and intrinsic Si show zero transparency, silicon, with generated excess carriers as well as doped Si, show high transparency up to 4 µm and 6 µm respectively.

When studying the transmission through a metallic film perforated with subwavelength holes array, usually a normalization of the transmission to both the input field as well as the hole area ratio is utilized. Such normalized transmission is called normalized-to-area transmission and is calculated as ${P}_{T-Norm}=\frac{{P}_{T}}{{P}_{i}\times \frac{{A}_{hole}}{{A}_{cell}}}$ ^1,3,7,9,12^ where ${P}_{T}$ is the actual transmitted power through the perforated thin film without normalization, ${P}_{i}$ is the input power, *A*_*hole*_ and *A*_*cell*_ are the hole and cell areas respectively. For example, if we have a hole area of 30% from the total cell area, it is expected, if there is no EOT, that the transmission would be 30% of the input field (i.e. $\frac{{P}_{T}}{{P}_{i}}=0.3$ and ${P}_{T-Norm}=\frac{{P}_{T}}{{P}_{i}\times \frac{{A}_{hole}}{{A}_{cell}}}=1$ indicating there is no extraordinary transmission). It is worth mentioning that such metric used heavily in literature ^1,3,7,9,12^, is a highly conservative metric for recognizing EOT through subwavelength holes. That is because a value less than or equal to one for ${P}_{T-Norm}$ is regarded as ordinary transmission in such literature while in reality subwavelength holes would not transmit all the light incident on them due to the diffraction limit according to Bethe’s theory and an ordinary transmission value would be much less than one. It was intentional, in the referenced literature, to use such a highly conservative metric to provide a healthy margin of safety when recognizing EOT. However, while this normalization would be a valid metric when used with real metallic films such as silver and gold which are opaque in the spectral range of interest, it would not be valid for thin-film materials that are semi-transparent at the designated thickness of the perforated thin film. To identify the transparency level and spectral range of the materials being utilized for the thin film, the Transmittance, which is the fraction of incident electromagnetic power that is transmitted through a sample, was calculated for a solid thin film with a thickness of 0.3 µm ($Transmittance={e}^{-(4\pi fk/c)d}$ where k is the imaginary part of the refractive index, d is the solid film thickness, c is the speed of light and f is the frequency). This was performed for Phosphorus doped Si with a doping concentration of 10^20^ cm^−3^, Si with generated excess carrier concentration of 10^20^ cm^−3^, intrinsic crystalline Si, and silver as shown in Fig. 3. From Fig. 3, Si with doping or generated excess carriers shows transparency in mid-IR. While maintaining the same conservative nature of the aforementioned transmission metric, a generalization is introduced in (1) to represent the expected transmission through a thin film perforated with an array of holes, no matter if the material of the thin film is semi-transparent or opaque. In (1), the expected transmitted power in case there is no EOT is calculated and then used in normalizing the actual transmitted power. ${P}_{T-Norm-expected}$ is the expected transmitted power normalized to input and area, ${P}_{T-solid}$ is the transmitted power through a solid thin film without normalization. The first term in ${P}_{T-Norm-expected}$ is $\frac{{P}_{T-solid}}{{P}_{i}}\times \left(1-\frac{{A}_{hole}}{{A}_{cell}}\right)$ which represents the expected normalized transmission through the region of the film area that is not occupied by the hole. Such region occupies a fraction of $\left(1-\frac{{A}_{hole}}{{A}_{cell}}\right)$ from the overall area. While the second term, which is $\frac{{A}_{hole}}{{A}_{cell}}$ represents the expected normalized transmission through the hole region. Since the hole region is filled with the same material as the surrounding material which the EM source already placed in, the normalized-to-input transmission through the hole is one. This is the reason the term $\frac{{A}_{hole}}{{A}_{cell}}$ is only multiplied by one. To demonstrate the semantics of such metric, let’s assume for example that the hole area is 40% of the cell area in a semi-transparent film. Since $\frac{{P}_{T-solid}}{{P}_{i}}$ represents the normalized to input transmission through the semitransparent film if there is no hole in it, then the expected transmission is composed of 60% of $\frac{{P}_{T-solid}}{{P}_{i}}$ and 40% of the normalized input field (which is unity) going through the hole. By normalizing the actual transmission to the input and the expected normalized transmission, we get the generalized normalized-to-area transmission metric. Such a metric will be reduced to the normalized-to-area metric used in literature for opaque thin films, as for such opaque films the ${P}_{T-Norm-expected}$ will only be $\frac{{A}_{hole}}{{A}_{cell}}$ since $\frac{{P}_{T-solid}}{{P}_{i}}$ would be zero.

1 $${P}_{T-Norm-expected}=\frac{{P}_{T-solid}}{{P}_{i}}\times \left(1-\frac{{A}_{hole}}{{A}_{cell}}\right)+\frac{{A}_{hole}}{{A}_{cell}}$$

$${P}_{T-Norm}=\frac{{P}_{T}}{{P}_{i}\times {P}_{T-Norm-expected}}$$

**Figure 4:**
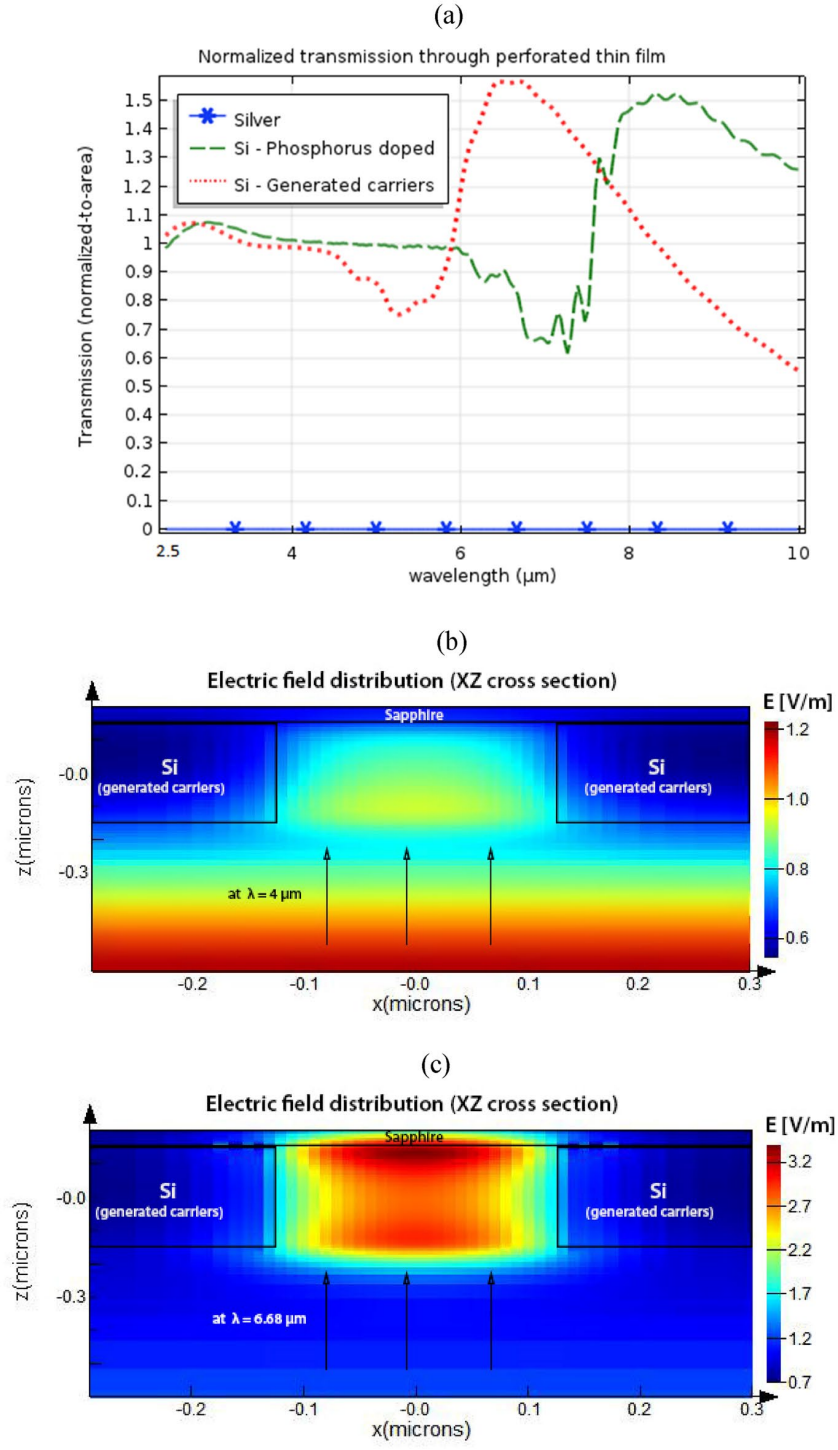

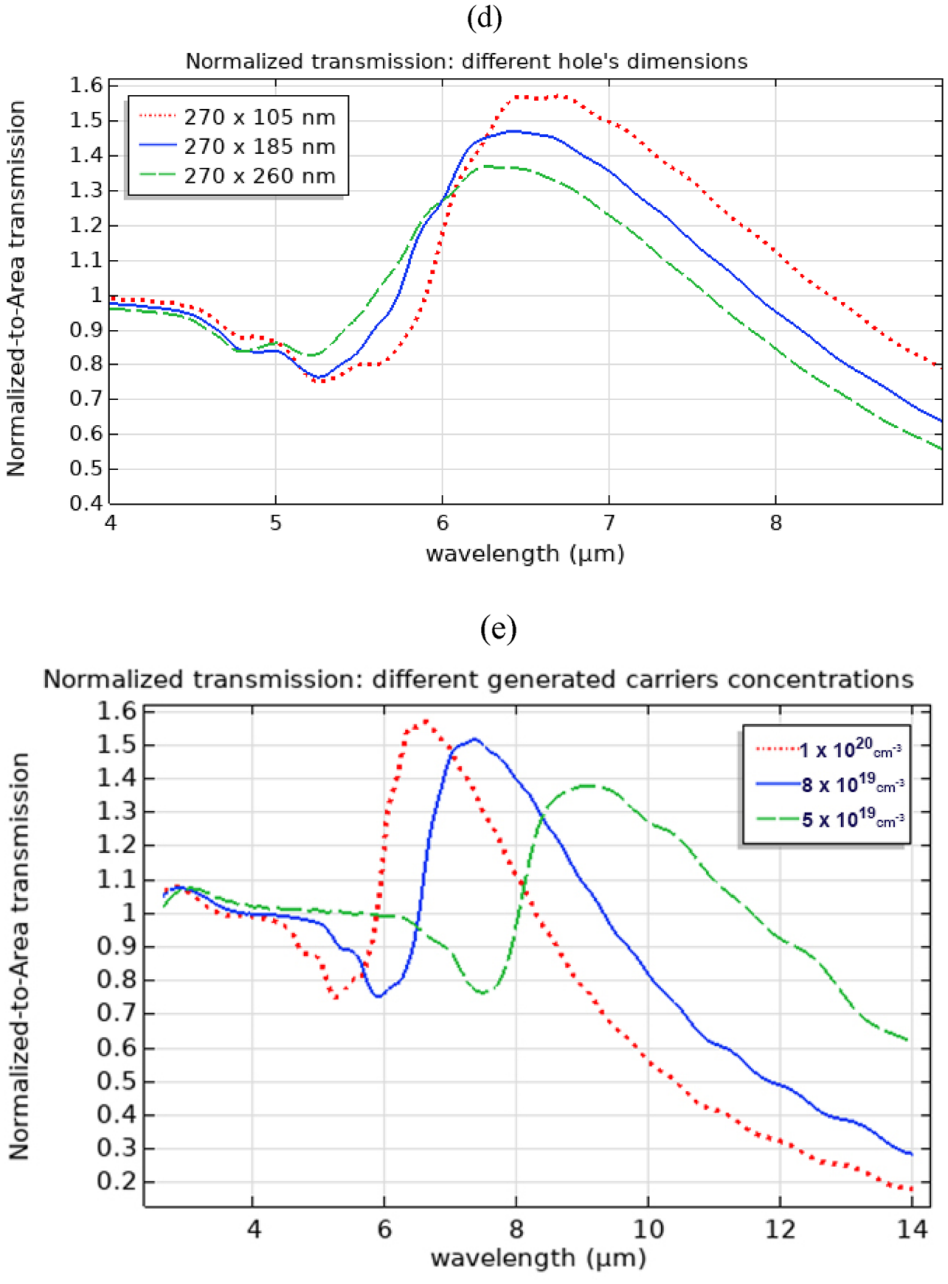
(**a**) Normalized to area transmission based on (1) through a perforated thin film with a periodic array of holes over a sapphire substrate. A comparison between different film materials of silver (blue-asterisk), Doped Si with 10^20^ cm^−3^ phosphorus doping concentration (green-dashed), and Silicon with generated excess carrier concentration of 10^20^ cm^−3^ (red-dotted) is shown over a spectral range from 2.5 to 10 µm. As expected Silver does not show any EOT in such mid-IR spectral range (Silver EOT peak should be around 900 nm as previously observed in the literature ^3,9^), while Si with generated excess carriers has a mid-IR EOT peak reaching 157% around 6.68 µm wavelength. The phosphorus-doped Si case shows transmission enhancement of 152% around 8.6 µm wavelength. (**b**) and (**c**) show the electric field distribution at 4.0 µm and 6.68 µm wavelengths respectively for the case of Si perforated thin film with generated excess carriers. The field distribution in (**b**) represents the case when there is no EOT, while (**c**) shows the field distribution in the case of EOT. The material of the subwavelength hole as well as the background material in (**a**), (**b**), and (**c**) was Air. The arrows in (**b**) and (**c**) represent the propagation direction. (**d**) shows the normalized-to-area transmission for different holes dimensions while maintaining the same hole width of 270 nm, and the same cell dimensions (600 × 600 nm). As the hole height increases, a blue shift in the EOT wavelength and lower enhanced transmission occurred. This trend conforms to the experimental results found in the silver film case ^56^. In (**e**), the effect of varying the generated excess carriers’ concentration on the normalized-to-area transmission is shown. While maintaining all other relevant parameters constant (i.e. cell and hole dimensions, background material, incident wave, substrate, etc.), the concentration cases of 10^20^, 8 × 10^19^, and 5 × 10^19^ cm^−3^ generated carriers was chosen for this figure as they caused EOT in the spectral range of interest (3–10 µm). Reducing the generated carrier’s concentration, caused a redshift in the EOT peak wavelength and a lower enhanced transmission.

The transmission, normalized based on (1), was studied for thin films made from Silver, phosphorus-doped Si, and Si with generated excess carriers. The structure design and dimensions were given in the previous section. Figure 4.a. shows a comparison among the normalized-to-area transmission computed for different film materials while the holes and background material were air. The spectral range of interest is 2.5 µm to 10 µm. In the silver case, the normalized transmission was in the order of 10^–4^ instead of one conforming to what was mentioned earlier about the conservative nature of the normalized to area transmission metric in general as it overestimates the expected transmission in subwavelength holes. As expected in this range, a perforated silver film would not generate EOT (blue-asterisk curve). The peak of EOT for perforated silver films with comparable dimension specifications is known to be around a wavelength of 900 nm ^3,9^. In the case of Si with generated excess carriers of 10^20^ cm^−3^ concentration, an EOT peak of 157% was observed in mid-IR around 6.68 (red-dotted curve). The phosphorus-doped Si case (green-dashed curve) showed transmission enhancement of 152% around 8.6 µm wavelength indicating a redshift in the EOT peak conforming to the redshift in the respective material dispersion shown in Fig. 2. In Figs. 4.b. and 4.c., a cross-section view, parallel to the propagation direction, showed the electric field distribution inside the subwavelength hole filled with Air with a thin film made of Si with 10^20^ cm^−3^ generated carriers. The case of ordinary transmission was shown in Fig. 4.b. as the wavelength was 4 µm and the normalized-to-area transmission is unity at such wavelength. Figure 4.c. shows the case of EOT transmission at 6.68 µm. The electric field distribution, in this case, shows an increase in the field intensity near the exit of the subwavelength hole. To probe the sensitivity of the transmission to different hole dimensions, Fig. 4.d shows the computed normalized-to-area transmission for different hole height values, specifically, 270 × 185, and 270 × 260 nm in addition to the 270 × 105 nm dimensions. This is analogous to the experiment performed on perforated silver film ^56^. Fixed cell dimensions of 600 × 600 nm were adopted in all cases. As the hole height increased, a blue shift in the EOT wavelength and lower enhanced transmission occurred. This behavior conforms to the experimental results found in the silver film case ^56^. In Fig. 4.e., the effect of varying the generated excess carriers’ concentration on the normalized-to-area transmission is shown. While maintaining all other relevant parameters constant (i.e. cell and hole dimensions, background material, incident wave, substrate, etc.), the concentration cases of 10^20^, 8 × 10^19^ and 5 × 10^19^ cm^−3^ generated carriers was chosen as they caused EOT in the spectral range of interest (3–10 µm). Reducing the generated carrier’s concentration, caused a redshift in the EOT peak wavelength and a lower enhanced transmission. Such a redshift in EOT wavelength was due to a similar redshift found in their respective material dispersion and the wavelength at which the permittivity value crosses from positive to negative.

The physical explanation behind EOT through a perforated thin film of plasmonic metal such as silver was given in the literature ^1,3,9,11,14,57^. In summary, the origin of the EOT resonance is the coupling between leaky surface modes on the metal–dielectric interface and the incident radiation. Such coupling is typically not possible on flat non-perforated thin metal film due to the lack of conservation of energy and momentum simultaneously. However, for perforated thin metal film, the periodic array of holes relaxes the momentum conservation condition and changes the characteristics of the surface modes. Hence, allows the coupling between the incident radiation and the surface modes to exist. Due to this, a large portion of the radiation usually got reflected from the metal surface is now being transmitted through the holes. The transmission through holes is enhanced even more due to the re-illumination of the holes by the existing surface modes which causes an additional buildup of transmission resonances^3^.

**Figure 5:**
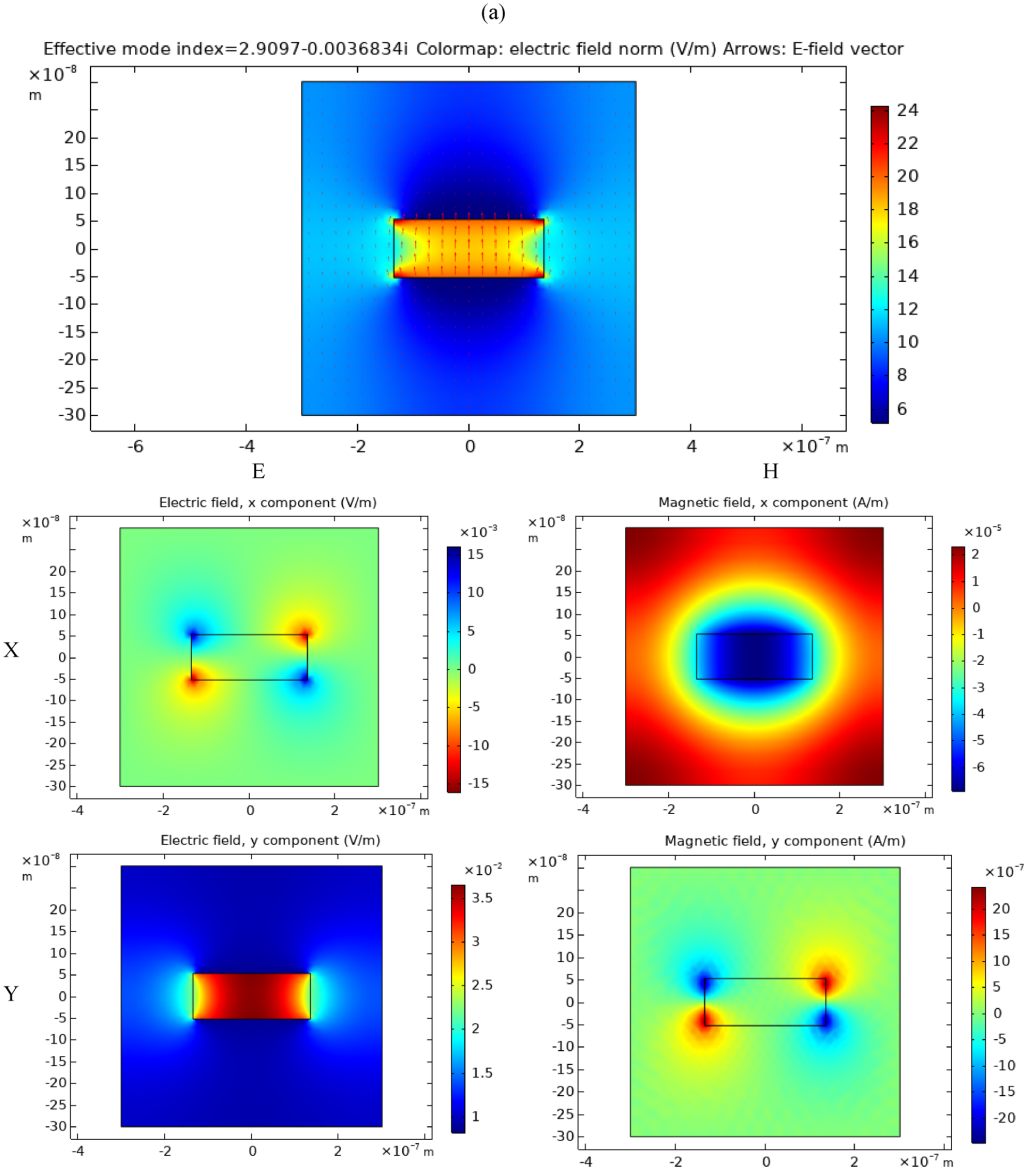

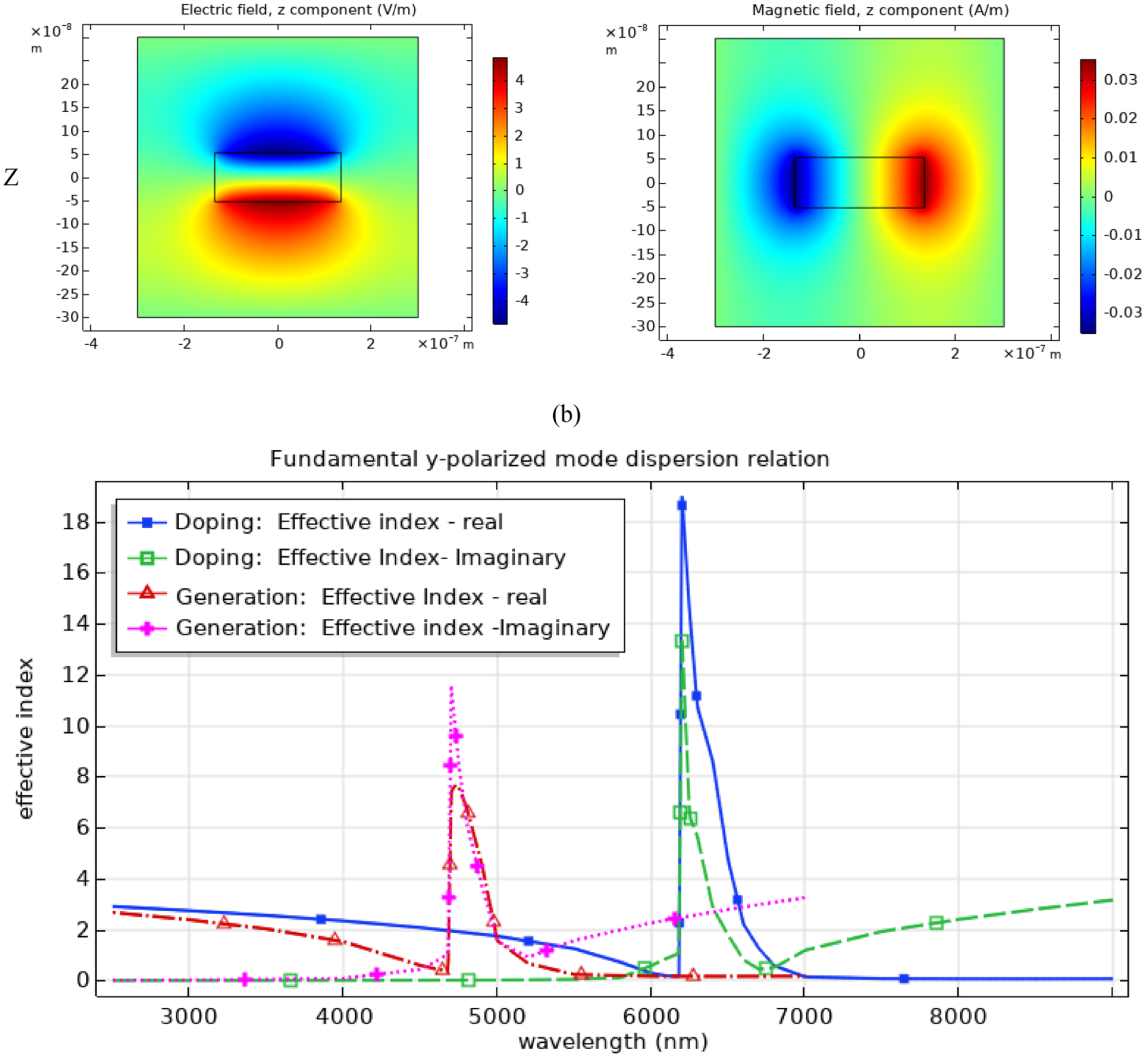
Fundamental y-polarized (**a**) mode profile and (**b**) dispersion relation in the case of doped Si perforated thin film with 10^20^ cm^−3^ phosphorus doping concentration (blue-solid and green-dashed curves) and the case of generated carriers with 10^20^ cm^−3^ concentration (red-dot-dashed and pink-dotted curves). The dispersion curves were found mimicking in their shape their counterparts for rectangular hollow waveguide in silver film, yet red-shifted to mid-IR ^58,59^. The cutoff wavelength is found to be around 5.5 µm and 7 µm for the cases of generated carriers and doping respectively. This shows that EOT peaks happen at larger wavelength than the cutoff wavelength which conforms to the literature studying relations between the fundamental mode cutoff and EOT in silver perforated thin films with subwavelength hole ^9,11^. The modal analysis performed for the structure dimensions shown in Fig. 1 with periodic boundary conditions.

It was also identified in literature that for perforated silver thin film with subwavelength holes, the wavelength at which EOT occurs is larger than the cutoff wavelength of the fundamental mode ^9,11^. To validate the same in case of perforated Si thin film with excess carriers due to doping or generation, modal analysis was performed in both cases and the dispersion curves were identified for the fundamental y-polarized mode. The modal analysis was performed for the structure dimensions shown in Fig. 1 with periodic boundary conditions. As shown in Fig. 5, the dispersion curves were found mimicking in their shape their counterparts for rectangular hollow waveguide in silver film, yet red-shifted to mid-IR ^58,59^. The cutoff wavelength is found to be around 5.5 µm and 7 µm for the cases of generated carriers and doping respectively. Since the EOT peak was at 6.68 µm and 8.6 µm for the cases of generated carriers and doping respectively (Fig. 4), it is clear that the relation between the EOT peak wavelength and the cutoff wavelength in the Si perforated thin film case is analogous to its counterpart in the case of metal films.

**Figure 6:**
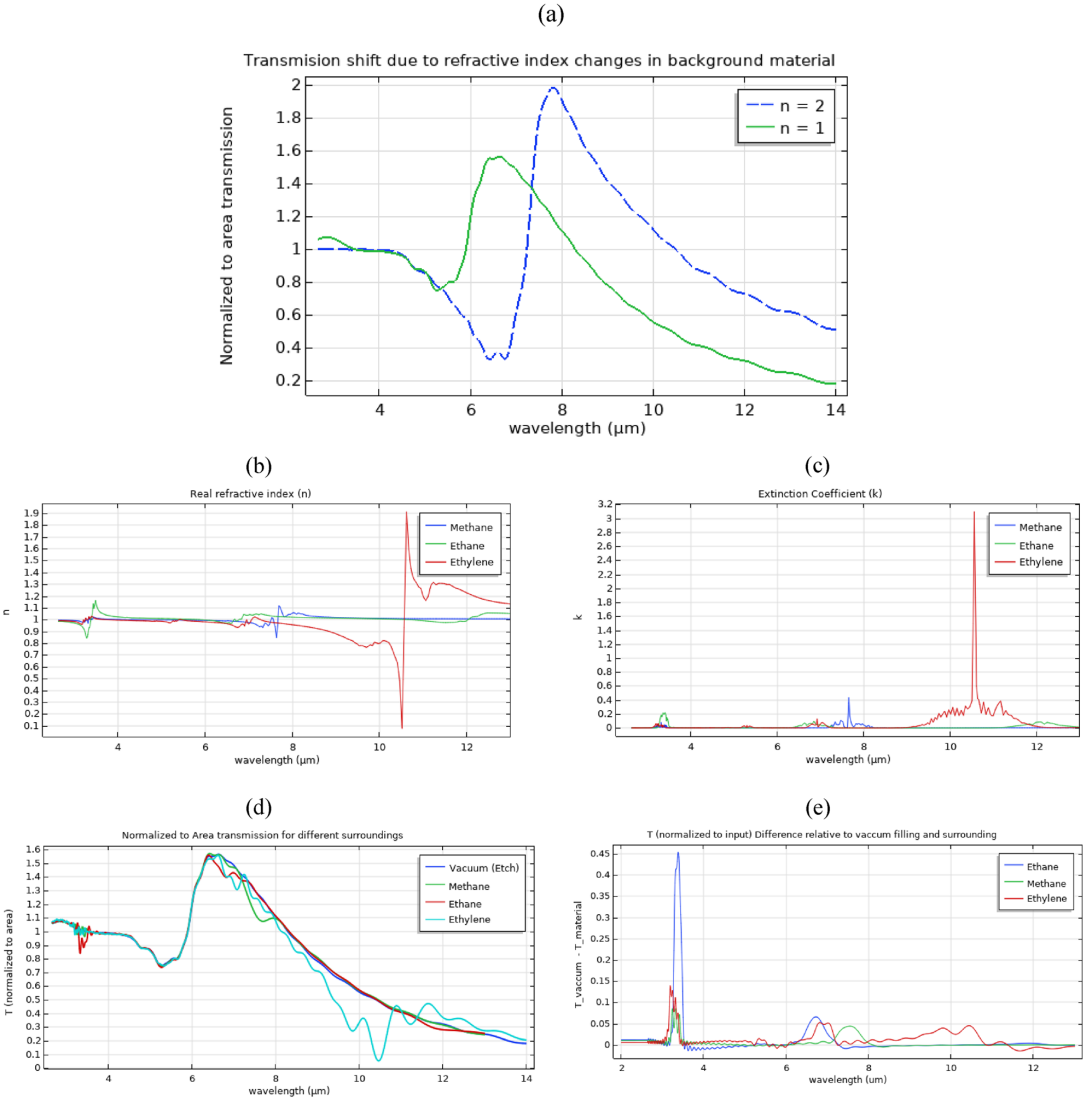
Analysis of the sensitivity of the calculated transmission to holes and surroundings material in a perforated Si thin film with generated excess carriers of 10^20^ cm^−3^ concentration on a Sapphire substrate. (**a**) shows a redshift of 1170 nm in the transmission peak for 1 Refractive Index Unit (RIU) increase (i.e. from 1 to 2) in the holes filling and background material’s index indicating a sensitivity of ~ 1170 nm/RIU. (**b**) and (**c**) are the real and imaginary components of the complex refractive indices of Methane, Ethane, and Ethylene generated from their NIST transmission data ^55^. In (**d**), normalized to area transmission through the perforated thin film for different surrounding materials and holes fillings are shown. Also as shown in (**e**), the differences in normalized (to input) transmission between the vacuum surroundings case and the case of Ethane, Methane, and Ethylene surroundings present more distinct fingerprints for recognizing the respective surrounding materials.

The sensitivity of the transmission to holes and surroundings material was analyzed to investigate EOT potential for sensing applications in Si-based structures. The case of perforated Si thin film with generated carriers of 10^20^ cm^−3^ was selected for such analysis. Figure 6.a shows a redshift of 1170 nm in the transmission peak for 1 Refractive Index Unit (RIU) increase (i.e. from 1 to 2) in the holes filling and background material’s index indicating a sensitivity of ~ 1170 nm/RIU. To study the potential of using EOT in sensing various gases with similar real refractive indices yet with different absorption fingerprints in mid-IR, Fig. 6.d shows the normalized-to-area transmission in the case of Ethane, Methane, and Ethylene used as the holes material and surroundings of the structure. Their complex refractive indices was calculated using Kramers–Kronig relations ^54^ from the transmission data with 5 cm path length. The transmission data was provided by NIST chemistry web book ^55^. As can be noticed from Fig. 6.b and 6.c, the complex refractive indices of the aforementioned materials are almost the same (approximately 1 and 0 for the real and the imaginary parts respectively) except for spikes due to the existence of absorption peaks in their transmission analysis in mid-IR as expected. Hence, as shown in Fig. 6.d, it is not expected to observe a shift in the realized EOT transmission peaks usually caused by a large variations in the refractive indices of the surrounding material. However, by calculated the difference in the normalized-to-input transmission between each of the aforementioned materials and vacuum surrounding and filling, unique transmission fingerprint through the perforated film was observed for each gas as shown in Fig. 6.e.

## Conclusions

A study was performed for the transmission through a thin film perforated with an array of subwavelength holes. For the first time, Si with a high concentration of excess carriers was targeted as the material of the thin film instead of plasmonic metals such as silver or gold. Both cases of doping and excess carriers’ generation were studied with a concentration of 10^20^ cm^−3^. Careful consideration was taken of the differences in the optical response between doped Si and silicon with induced excess carriers. A curve-fitting model was crafted based on experimental results ^39,40^ to provide an accurate optical response model for intrinsic Si in the mid-IR range (from 2.5 µm to 22 µm) (Fig. 2, solid blue curve). This model captures the inter-band transitions and absorption peaks in mid-IR observed in intrinsic Si ^40^. An index perturbation approach was utilized ^41–43^ to incorporate the plasma dispersion effect introduced by doping or optical generation of excess carriers into the overall refractive index of the material. The material dispersion in both cases was modeled and plotted.

The normalization to area transmission metric utilized in the recent EOT research literature ^1,3,7,9,12^ was generalized to apply to semi-transparent thin films as well and allow for valid calculation of the normalized to area transmission through Si thin films with excess carriers. The transmittance curves of different thin-film materials at 300 nm thickness were compared indicating a semi-transparent spectral range from near to mid-IR in the case of silicon with excess carries.

The resulted normalized-to-area transmission through a perforated thin film was computed using full-wave simulation by utilizing an FDTD credible simulation package tool ^37^ to numerically solve Maxwell equations in 3D. Such normalized transmission was compared in the case of silver, doped Si, and Si with generated excess carriers in the mid-IR spectral range. An EOT was observed successfully in the results while using Si with excess carriers. Si, with generated carriers, showed stronger, blue-shifted EOT compared to the doping case. Such shift conforms to the material dispersion shift that existed between the doping and the generated excess carrier Si cases. The higher EOT value in the excess carrier case could be related to the existence of a high concentration of both electrons and holes with higher mobility due to the absence of donors or acceptor scattering. The case of Si with generated excess carriers had a mid-IR EOT peak reaching 157% around 6.68 µm wavelength. The phosphorus-doped Si case showed transmission enhancement of 152% around 8.6 µm wavelength. A cross-section of the electric field distribution showed a higher concentration of the field at the exit of the hole compared to the entrance. By increasing the height of the subwavelength holes, a lower and blue-shifted EOT occurred conforming to analogous experimental results of the silver film^56^. By reducing the concentration in the generated carriers’ case, a lower red-shifted EOT occurred as a result of a similar shift in the respective material dispersion. Modal analysis of the fundamental y-polarized mode of the rectangular subwavelength hole showed dispersion curves mimicking in their shape their counterparts for rectangular subwavelength hollow waveguide in silver film, yet red-shifted to mid-IR ^58,59^. The cutoff wavelength was found to be around 5.5 µm and 7 µm for the cases of generated carriers and doping respectively. It was clear that the relation between the EOT peak wavelength and the cutoff wavelength in the Silicon perforated thin film case studied was analogous to its counterpart in the perforated silver thin film case in literature ^9,11^. To investigate the sensitivity of the basic structure if used as a sensor for the hole filling material as well as the surrounding material, the normalized transmission was analyzed showing a sensitivity of 1170 nm/RIU for perforated Si thin films with generated excess carriers. Methane, ethane, and ethylene gases had their complex refractive indices in the mid-IR calculated using Kramers–Kronig relations ^54^ from NIST transmission data with 5 cm path length ^55^. Although they showed very similar complex refractive indices values, it was shown that they can be differentiated by calculated the difference in the normalized-to-input transmission between each of the aforementioned materials and vacuum surrounding and filling, such difference showed a unique transmission fingerprint through the perforated film for each gas. The possibility of tuning the generation of excess carriers would also offer the flexibility of tuning the performance and operating range of EOT-based devices for various applications.
